# Red blood cell distribution width as a prognostic marker of mortality in patients on chronic dialysis: a single center, prospective longitudinal study

**DOI:** 10.3325/cmj.2013.54.25

**Published:** 2013-02

**Authors:** Mario Sičaja, Mario Pehar, Lovorka Đerek, Boris Starčević, Vladimira Vuletić, Željko Romić, Velimir Božikov

**Affiliations:** 1 Department of Medicine, Dubrava University Hospital, Zagreb, Croatia; 2 Clinical Department for Laboratory Diagnostic, Dubrava University Hospital, Zagreb, Croatia; 3 Department of Neurology, Dubrava University Hospital, Zagreb, Croatia

## Abstract

**Aim:**

To determine if red cell distribution width (RDW) is associated with all-cause mortality in patients on chronic dialysis and to evaluate its prognostic value among validated prognostic biomarkers.

**Methods:**

This is a single center, prospective longitudinal study. At the time of inclusion in January 2011, all patients were physically examined and a routine blood analysis was performed. A sera sample was preserved for determination of NT-pro-brain natriuretic peptide (NT-pro-BNP) and eosinophil cationic protein. Carotid intima media thickness (IMT) was also measured. Following one year, all-cause mortality was evaluated.

**Results:**

Of 100 patients, 25 patients died during the follow-up period of one-year. Patients who died had significantly higher median [range] RDW levels (16.7% [14.3-19.5] vs 15.5% [13.2-19.7], *P* < 0.001. They had significantly higher Eastern Cooperative Oncology Group (ECOG) performance status (4 [2-4] vs 2 [1-4], *P* < 0.001), increased intima-media thickness (IMT) (0.71 [0.47-1.25] vs 0.63 [0.31-1.55], *P* = 0.011), increased NT-pro-BNP levels (8300 [1108-35000] vs 4837 [413-35000], *P* = 0.043), and increased C-reactive protein (CRP) levels (11.6 [1.3-154.2] vs 4.9 [0.4-92.9], *P* < 0.001). For each 1% point increase in RDW level as a continuous variable, one-year all cause mortality risk was increased by 54% in univariate Cox proportional hazard analysis. In the final model, when RDW was entered as a categorical variable, mortality risk was significantly increased (hazard ratio, 5.15, 95% confidence interval, 2.33 to 11.36) and patients with RDW levels above 15.75% had significantly shorter survival time (Log rank *P* < 0.001) than others.

**Conclusions:**

RDW could be an additive predictor for all-cause mortality in patients on chronic dialysis. Furthermore, RDW combined with sound clinical judgment improves identification of patients who are at increased risk compared to RDW alone.

In patients on chronic dialysis, the prevalence of cardiovascular disease is very high, and among patients with chronic renal failure atherosclerosis and cardiovascular diseases are the leading cause of morbidity and mortality (1,2). Recent investigations of atherosclerosis have focused on inflammation, emphasizing the importance of endothelial dysfunction and inflammatory biomarkers interaction, suggesting that a biomarker such as C-reactive protein plays a key role in promoting atherosclerosis process and endothelial cell activation and inflammation (3,4). C-reactive protein and NT-pro-brain natriuretic peptide (NT-pro-BNP) are strong and validated prognostic biomarkers, which are considered as gold standard in patient risk assessment and survival analysis (5). Also, recent studies have identified eosinophilic cationic protein (ECP) as a biomarker of coronary atherosclerosis (6). It has been stated that ECP serum concentration is proportional to the growth of atherosclerotic plaque in the coronary vessels (6).

Several studies have identified red blood cell distribution width (RDW) as a strong and independent predictor of morbidity and mortality in general population (7,8), as well in different groups of patients with morbidities such as acute or chronic heart failure, cardiac arrest, pulmonary embolism, acute coronary syndrome, and even community acquired pneumonia (9-13). Furthermore, RDW has been identified as independent short- and long-term prognostic marker in intensive care unit patients, which significantly improves risk stratification of simplified acute physiology score (SAPS) (14). It is defined as a measure of variability in size of circulation erythrocytes and has traditionally played a role in the differential diagnosis of anemia (10). In everyday clinical practice, it is an automatically measured index, which is calculated by dividing standard deviation (SD) of red blood cells volume by mean corpuscular volume (MCV) and multiplying by 100 to express the results as percentage (10,15). Recently, it has been demonstrated that RDW could be an additive predictor for all-cause mortality in patients with acute renal failure treated with continuous renal replacement therapy (16). However, there are no data among patients with chronic renal failure treated with maintenance dialysis. Therefore, we aimed to investigate whether RDW was associated with all-cause mortality in patients on chronic dialysis and whether it would provide meaningful prognostic value among validated prognostic biomarkers.

## Methods

### Patients

This prospective longitudinal study was conducted in a hemodialysis department of a single tertiary academic hospital with approximately 115 patients on chronic hemodialysis who were screened for participation. All patients with chronic renal failure who were treated with maintenance hemodialysis at the dialysis unit (Department for Hemodialysis, University Hospital Dubrava) between December 2010 and January 2011, and who had been on hemodialysis for at least one year, were eligible for inclusion. Exclusion criteria were malignant disease, autoimmune disease, chronic immunosuppressive treatment, or recent surgical procedure. Finally in January 2011, 100 patients were included in the study cohort. All included individuals underwent detailed general examination with cardiovascular priority and were given a simple questionnaire (supplementary questionnaire) (web extra material 1) for evaluation of traditional risk factors. Body-mass index (BMI) was defined as weight (kg) per body surface (m^2^). Influence of dialysis was expressed as duration of dialysis in months and its performance as outcome Kt/V (K – dialyzer clearance of urea × t – dialysis time/V – volume of distribution of urea, approximately equal to patient's total body water), which was calculated by the Daugirdas method, based on the reduction in the serum urea concentration during dialysis (17). Patients’ daily living abilities were graded according to Eastern Cooperative Oncology Group performance status (ECOG) (18) ranging from 0-5, with 0 indicating that the patient is fully active and capable for everyday normal activity and 5 indicating that he or she is dead (18). The intima-media thickness (IMT) of both carotid arteries was measured ultrasonographically with an Aloka 5500 Prosound machine (Hitachi Aloka, Tokyo, Japan) using a 7.5 MHz high-resolution probe. IMT was defined according to the Mannheim Carotid Intima-Media Thickness Consensus (19). The patient follow-up was performed by means of telephone calls and personal interviews. The study was approved by the local ethics committee and all patients gave informed consent. The studied end-point was all-cause mortality. We would like to emphasize that all patients included in the study were treated in accordance with standardized protocols for their disease/condition and that their inclusion in this study had no effect on their treatment, care provided, or the final outcome.

### Laboratory assessment

Complete blood count (CBC) (including RDW calculation) was determined from whole blood with K2EDTA as an anticoagulant on Advia 2120i analyzer (Siemens Healthcare Diagnostics, Tarrytown, NY, USA). The reference range of RDW for normal population in our laboratory is between 9%-15%, but we used the median value because our analysis was performed in dialysis population, which is different from normal population. The concentrations of ECP, NT-pro-BNP, high sensitive C-reactive protein (hs-CRP), creatinine, albumin, total cholesterol, iron, and unsaturated iron binding capacity (UIBC) were determined in the sera samples collected from all the participants. The sera were obtained after centrifugation at 1370 × g for 15 minutes in a 35 R Rotina Hettich centrifuge (Tuttlingen, Germany), and then stored at -80°C until analysis. ECP was measured using fluoroimmunoassay method on Phadia 100 analyzer (Phadia AB, Uppsala, Sweden). The method includes the reaction of anti-ECP covalently coupled to ImmunoCAP with the ECP in the patient sample. After washing, enzyme labeled antibodies to ECP were added to form a complex, and after incubation, the bound complex was incubated with a developing agent. The measured fluorescence of the eluate was proportional to the ECP concentration in the sample (20). The concentration of NT-proBNP was determined using electrochemiluminiescence immunoassay on Cobas e411 analyzer (Roche Diagnostics GmbH, Mannheim, Germany) (21). The method principle is a sandwich principle where antigen in the sample, biotinylated monoclonal anti-NT-proBNP specific antibody, and a monoclonal NT-proBNP-specific antibody labeled with ruthenium complex react and form a sandwich complex. Afterwards, the complex bounds to streptavidin-coated microparticles, which are magnetically captured onto the surface of the electrode. Application of voltage to the electrode induces chemiluminiscent emission, which is measured by a photomultiplier.

The concentration of hsCRP was determined using immunoturbidimetric method on AU 2700 plus analyzer (Beckman-Coulter, Tokyo, Japan). In brief, CRP reacts with antibodies to human CRP latex particles, forming insoluble aggregates. The absorption of generated aggregates is proportional to the concentration of CRP in the sample. The method was calibrated with Latex CRP Calibrator Set High Sensitive (ODCO27) with five different calibrator concentrations for low sensitive area (22).

Creatinine, total cholesterol, albumin, iron, and UIBC were determined on AU 2700 plus analyzer (Beckman-Coulter). Creatinine and total cholesterol were determined using enzymatic color tests. Albumin, iron, and UIBC were determined using photometric color tests.

### Statistical analysis

The study population was divided into two groups according to the median of red cell distribution width (RDW) in order to facilitate the clinical application of our results. A similar study design was used in previously published studies (10,16). All variables were tested for normal distribution by the Kolmogorov-Smirnov test. Summary statistics for the continuous variables were presented as mean± SD or median with range, and comparisons between the two groups were preformed with the Mann-Whitney U test. Categorical data were expressed as number (N), and comparisons between categorical data in Table 1 were preformed with χ^2^ test. Correlations between RDW and other continuous variables were tested using Pearson test or the Spearman correlation, as appropriate. Kaplan-Meier survival curves were drawn and Log-rank values were calculated to assess their statistical significance. Prognostic variables for mortality were analyzed by using the univariate Cox proportional hazards model, and variables with *P*-value <0.1 in univariate analysis were used in the stepwise multivariate Cox proportional hazards model. The univariate and multivariate Cox regression analysis results are presented as hazard ratios (HR) and 95% confidence intervals. The discrimination of RDW for one-year all-cause mortality was evaluated using the area under the receiver operating characteristic (ROC) curve. In addition, we divided the patients into grade 1 or 2 group depending on RDW value (RDW group 1 consisted of patients who had RDW below the median RDW and RDW group 2 consisted of patients who had RDW above the median RDW). RDW group 1 was given score 1 and RDW group 2 was given score 2, and the score was subsequently added to baseline ECOG score. AUC was calculated for RDW levels, ECOG score, and ECOG score plus graded RDW score. The optimal cut off point for ROC curves was selected for maximizing the sensitivity and specificity of the selected values. *P*-value of <0.05 was considered statistically significant. MedCalc, version 11.4.2.0 (Ostend, Belgium) and JMP, version 9.0.2 software (Cary, NC, USA) were used.

**Table 1:** Baseline characteristics (demographics, history, clinical and laboratory parameters) of patients on hemodialysis, divided according to red blood cell distribution width (RDW) (median)

Variable	RDW≤15.75 (n = 50)	RDW>15.75 (n = 50)	P*
Age (years), mean±SD^†^	66.4 ± 14.2	67.7 ± 14	0.484
Male sex, N	23	29	0.926
Body mass index (kg/m^2^), mean±SD	25.9 ± 4.8	25.5 ± 5.3	0.539
History of arterial hypertension, N	41	44	0.416
History of diabetes mellitus, N	14	21	0.148
History of coronary artery disease, N	14	10	0.3595
History of stroke/ transitory ischemic attack, N	1	7	0.037
Erythropoietin therapy, N	46	44	0.525
Duration of dialysis (months), median (range)	36 (6-180)	29 (6-360)	0.390
Kt/V, mean±SD	1.37 ± 0.21	1.3 ± 0.24	0.068
Eastern Cooperative Oncology Group performance status, median (range)	2 (1-4)	2.5 (1-4)	0.105
Atrial fibrillation, N	7	14	0.093
Carotid intima-media thickness (mm), mean±SD	0.65 ± 0.17	0.71 ± 0.20	0.148
ACE/AT II blockers at presentation, N	18	20	0.685
Beta-blocker at presentation, N	17	19	0.681
Calcium-blocker at presentation, N	23	32	0.074
Statins at presentation, N	12	17	0.279
Proton pump inhibitor at presentation, N	12	19	0.137
White blood cells (cells ×10^9^/L), mean±SD	7.02 ± 2.04	7.08 ± 1.93	0.796
Hemoglobin (g/L), mean±SD	114 ± 15	109 ± 17	0.196
Mean corpuscular volume (fL), mean±SD	96 ± 4	96 ± 6	0.890
Creatinin (mmol/L), mean±SD	825 ± 196	809 ± 254	0.590
hs-C-reactive protein (mg/L), median (range)	5.725 (0.6-92.9)	7.850 (0.4-154.2)	0.125
nt-pro-brain natriuretic peptide (pg/mL), median (range)	4671 (413-35000)	6633 (466-35000)	0.131
Serum albumin (g/L), mean±SD	38 ± 3	36 ± 4	0.147
Total cholesterol (mmol/L), mean±SD	4.33 ± 1.01	4.45 ± 1.34	0.920
Iron (µmol/L), mean±SD	12.6 ± 4.5	11.6 ± 5.1	0.359
Total iron binding capacity (µmol/L), mean±SD	35.1 ± 5.6	34.8 ± 7.9	0.793
Eosinophilic cationic protein (µg/L), mean±SD	15.69 ± 10.06	18.33 ± 17.26	0.912

*Mann-Whitney test was predominately used, except for calculation of difference in sex, wher χ^2^ test was used. *P* < 0.05 is considered as significant.

^†^SD – standard deviation.

## Results

### Population characteristics

The study population consisted of 100 patients who were stratified at the time of inclusion by median RDW of 15.75 (Table 1). Patients with an elevated RDW had higher incidence of stroke/TIA (*P* = 0.0371), but there were no significant differences between the groups in other variables. Fifty two patients were men and the median age of all patients was 72 years (interquartile range 28-93). RDW ranged from 13.2 to 19.7; with a mean value of 15.9 ± 1.4.

### Association between RDW values and other parameters

There were no significant correlations between RDW and RBC indices, such as hemoglobin and MCV (r = -0.0896, *P* = 0.375; r = 0.0491, *P* = 0.627). Correlation between RDW and serum albumin, atrial fibrillation, stroke history, serum iron, and beta-blocker usage was significant but marginal (r = -0.282, *P* = 0.038; r = 0.208, *P* = 0.038; r = 0.227, *P* = 0.023, r = -0.240, *P* = 0.016, respectively). We found no correlations between RDW and WBC, total cholesterol, serum creatinine, serum ECP, hs-CRP, TIBC, KT/V, ECOG status, and IMT.

### Survival vs non-survival group

**Figure 1:**
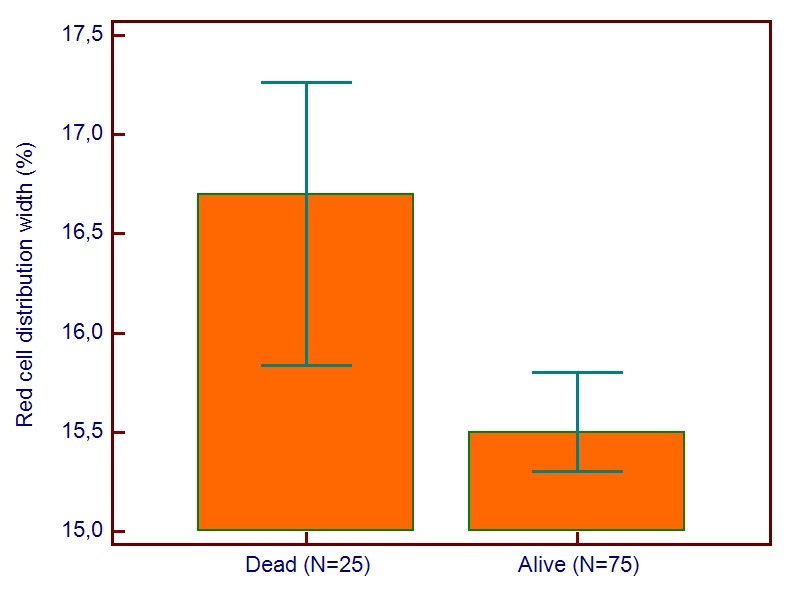
Red blood cell distribution width (RDW) measures as a function of mortality at one year of follow-up (data presented as median of RDW with 95% confidence interval for median).

A total of 25 patients died during the follow-up period of one-year. At inclusion time, patients who died had significantly higher RDW levels (16.7% [14.3-19.5] vs 15.5% [13.2-19.7], *P* < 0.001, Figure 1) and lower hemoglobin levels (107 [61-138] vs 115 [65-138], *P* = 0.048). They also had significantly higher ECOG class (4 [2-4] vs 2 [1-4], *P* < 0.001), increased IMT (0.71 [0.47-1.25] vs 0.63 [0.31-1.55], *P* = 0.011), increased nt-pro-BNP concentrations (8300 [1108-35000] vs 4837 [413-35000], *P* = 0.043), and increased C-reactive protein concentrations (11.6 [1.3-154.2] vs 4.9 [0.4-92.9], *P* < 0.001).

### Risk analysis for all-cause mortality

**Figure 2:**
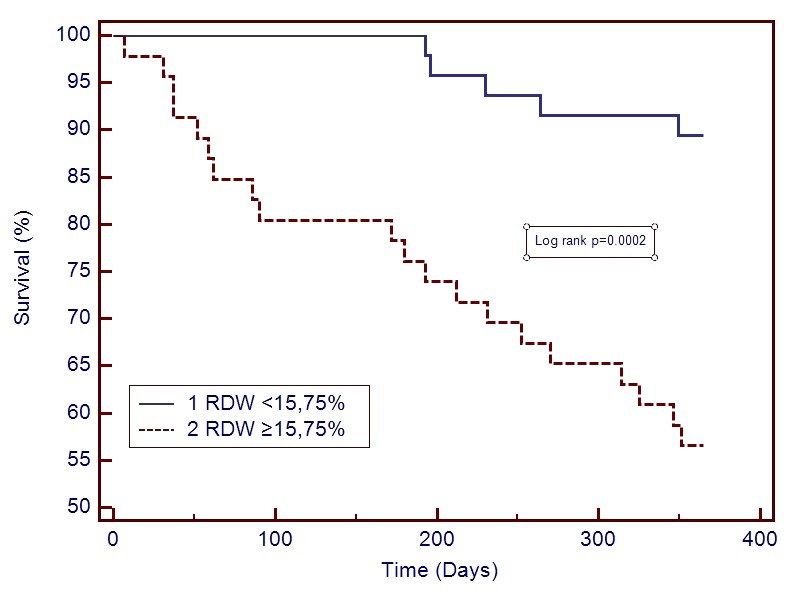
Kaplan-Meier survival curves according to red blood cell distribution width (RDW) values above or below the RDW median (15.75%).

For each 1% point increase in RDW value as a continuous variable, one-year all cause mortality risk was increased by 54% in univariate Cox proportional hazard analysis. In multivariate Cox proportional hazard model, in order to examine independent nature of RDW in prediction of all-cause mortality an adjustment for known co-morbidities and potent predictors was made. In the final calculation, a prognostic value of RDW was strong and independent (HR 1.5346, 95% CI, 1.1607 to 2.0290, *P* = 0.002), even after adjustment for co morbidities and previously known significant predictors for all-cause mortality at one year (Table 2). In the final model, when RDW was entered as categorized variable according to the median value of the study group, mortality risk was significantly increased (HR 5.15, 95% CI 2.33 to 11.36) and patients with RDW value above 15.75% had significantly lower survival time (Log rank *P* < 0.001) (Figure 2).

**Table 2:** Cox proportional hazards analysis for all-cause mortality at 12 mo

	Univariate		Multivariate	
	hazard ratio (95% confidence interval)	*P* (Cox proportional hazard analysis)	hazard ratio (95% confidence interval)	*P* (Cox proportional hazard analysis)
Age, years	1.0560 (1.0190-1.0945)	<0.001		NS*
Eastern Cooperative Oncology Group performance status	3.5347 (2.1569-5.7927)	<0.001	3.8624 (2.2769-6.5521)	<0.001
Kt/V	0.0454 (0.0065-0.3177)	<0.001	0.0189 (0.0023-0.1580)	<0.001
Atrial fibrillation	0.2640 (0.1201-0.5804)	<0.001		NS
Carotid intima-media thickness	21.2398 (2.9253-154.2142)	0.004		NS
Diabetes mellitus	2.2056 (1.0100-4.8165)	0.049		NS
Hemoglobin	0.9734 (0.9522-0.9951)	0.022		NS
Highly sensitive C-reactive protein	1.0177 (1.0076-1.0278)	0.005	1.0255 (1.0119-1.0392)	<0.001
NT-pro-brain natriuretic peptide	1 (1.0000-1.0001)	0.019		NS
Eosinophil cationic protein	1.0322 (1.0005-1.0648)	0.049		NS
Red cell distribution width	1.5409 (1.2181-1.9493)	<0.001	1.5346 (1.1607-2.0290)	0.002

*NS – not included in multivariate model due to limitations made by used statistic model.

**Figure 3:**
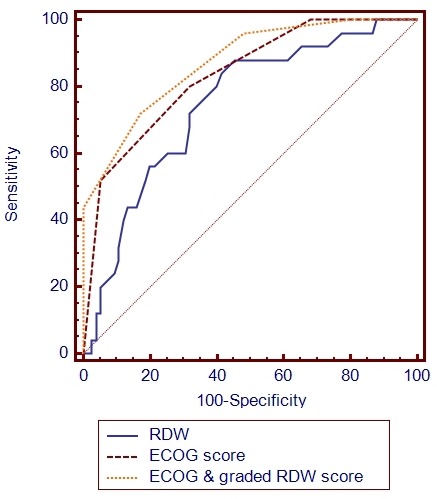
Additive prognostic value of red blood cell distribution width (RDW) in patients on chronic dialysis measured by comparison of area under the receiver operating characteristic curve (AUC) for 1) RDW alone, 2) Eastern Cooperative Oncology Group performance (ECOG) alone, and 3) ECOG plus graded RDW score.

The ROC curves using variables (RDW value, ECOG value, and ECOG value plus graded RDW score) are plotted in Figure 3. The area under the curve (AUC) of RDW value alone for one-year all-cause mortality was 0.745 (95% CI 0.648 to 0.827; optimal cut-off value at 15.5% with sensitivity 88.0% and specificity 54.6%, *P* < 0.001) and of ECOG status 0.834 (95% CI 0.746 to 0.901; optimal cut-off value at 2 with sensitivity 80.0% and specificity 68.0%, *P* < 0.001). In a pair-wise comparison of ROC curves, there was no significant difference between AUC of RDW and ECOG (95% CI -0.0496 to 0.228, *P* = 0.207), but adding graded RDW to ECOG status significantly improved prognostic performance of the RDW alone model (AUC 0.872, 95% CI 0.0176 to 0.237, *P* = 0.022).

## Discussion

Our study indicated that RDW value was an independent predictor of all-cause mortality in patients on chronic dialysis. This finding remained significant even in the presence of other powerful prognostic biomarkers such as NT-proBNP, hsCRP, or IMT, which are all validated prognostic parameters in chronic dialysis (23-25). When we divided patients into two groups according to the RDW median of 15.75%, the survival rate was significantly lower in the higher RDW group, but there were no significant differences between the groups in the tested variables except for stroke/TIA incidence. There was also an independent and additive effect of RDW in the assessment of survival in patients on chronic dialysis. Previous studies in general population and specific subgroups of patients found significant correlation between RDW and hemoglobin concentration, MCV, hsCRP, and WBC (7,10,25), while our study found no significant correlation between RDW and RBC indices, suggesting that pathogenesis of elevated RDW in patients on chronic dialysis was even more complex than in general population. In some studies performed on patients on dialysis, it was shown that RDW was associated with iron deficiency status (26), but our study showed RDW value to be independent of iron status.

An interesting finding was an increased incidence of stroke/TIA in the group with higher RDW. Similarly, Ani et al found that elevated RDW was associated with stroke occurrence and strongly predicted both cardiovascular and all-cause deaths in persons with known stroke (27), which could all indicate a possible role of RDW in the increased risk of cerebrovascular thrombosis (26,28). Our data on this subject are limited, and a specific study should be designed to test this finding. Although one could suspect that RDW is primary connected with atherosclerosis, or with ECP, which was presented as biomarker of coronary atherosclerosis, our results do not implicate that this biomarker is relevant for RDW values, suggesting a pathogenic pathway different from those of ECP (5).

ECOG score has been commonly used for years in patients with malignancy (18), but it can be applied for accurate assessment of daily living abilities in every patient. When we added graded RDW score to ECOG score, we significantly improved prognostic performance of the RDW alone model. Combining a simple clinical assessment tool, such as ECOG score, with RDW resulted in a very reliable prognostic tool, which can be applicable among patients in chronic dialysis in everyday clinical practice. These results are in line with those of Hunziker et al (14), who have proven that adding RDW to SAPS prognostic tool significantly improves prognostic reliability of SAPS score in identifying critically ill patients.

Correlation between RDW and albumin, atrial fibrillation, stroke history, and iron and beta-blocker usage was significant but marginal. A negative significant correlation of RDW with albumin and atrial fibrillation could be explained by a more frequent occurrence of both hypoalbuminemia and atrial fibrillation in patients who are in the chronic state of malnutrition (16). It has already been hypothesized that malnutrition could be one of the causes of increased RDW in patients on chronic dialysis (29).

Little is known about the mechanism by which elevated values of RDW are associated with increased mortality. Usually, RDW is elevated when there is increased red cell destruction, or what is more common, ineffective and increased red cell production, which are both prevalent in patients on dialysis (29). RDW may represent malnutrition, suppression of bone marrow production, or chronic inflammation (16). Although the mainstay of atherosclerosis pathogenesis and progression is chronic inflammation, it is highly unlikely that the relation of RDW to mortality risk is based only on the premise of chronic inflammation. Furthermore, it has been shown that the risk associated with RDW was not significantly diminished in participants with a low CRP compared with those with a high CRP level (14,16). Therefore, a final theory is based on oxidative stress association with RDW (14). Oxidative stress has been shown to increase anisocytosis by disrupting erythropoiesis and to alter blood cell membrane deformability and red blood cell circulation half-life, ultimately leading to increased RDW (14). The proposed mechanism includes ischemia and oxidative stress as a driving force that activates cellular systems that would reduce oxygen demand and physiologic processes that would improve tissue oxygen delivery, such as increased production and release of mature red cells into the peripheral bloodstream. Release of large immature red cells with poor oxygen-binding capacity, which results in an increased RDW, implies suboptimal response to oxidative stress. This has been offered as an explanation why the association between RDW and clinical outcome is independent of the severity of acute illness as well as the degree of inflammation (14). This hypothesis requires further investigation, but it seems that it could be also applied to our patient group. Whatever the reason may be, all of these conditions are common in patients on hemodialysis and are associated with unfavorable prognosis (30). Association of RDW with all-cause mortality indicates that not only deaths from cardiovascular diseases, but cancer and other causes are all connected to RDW, which is also supported by findings of a meta-analysis on older populations (8)

This study has several limitations. We did not evaluate fluctuations in RDW values and thus could not account for possible variation over time. Also, regardless of the prospective longitudinal design of the study, one year is a rather short follow-up and we would suggest a larger, multicenter study with longer follow-up to make definitive conclusions and evaluate our findings. Despite these limitations, a major strength of this study lies in its prospective design with good follow-up and low drop-out. What is more important, this study is based on real-life “every day dialysis patient” sample.

In conclusion, our study demonstrated that RDW could be an additive predictor for all-cause mortality in patients on chronic dialysis. Available literature data do not provide clear explanations for such a finding, but nevertheless RDW combined with sound clinical judgment, ie, ECOG score improves identification of patients with an increased risk compared to RDW model alone. Since RDW is a simple, inexpensive, and widely available test, these data may have significant clinical implications for assessing prognosis and choice of treatment in patients on chronic dialysis.

## Additional Material

Supplementary qustionnaire
