# Supplementary material for: Red blood cell distribution width as a prognostic marker of mortality in patients on chronic dialysis: a single center, prospective longitudinal study
Source: Croat Med J. 2013 Feb;54(1):25–32. doi: 10.3325/cmj.2013.54.25 (PMC3583391; doi:10.3325/cmj.2013.54.25)
Supplement: Supplementary qustionnaire [file CroatMedJ_54_s002.pdf]

Clinical Hospital Dubrava

Av.Gojka Šuška 6

Zagreb, Croatia

Clinical Questionnaire

**Clinical Hospital Dubrava**	Patient No. ________________	Date of examination ____________
Sex: ☐ Male ☐ Female		Year of birth *(YYYY)*: ___ ___ ___ ___<br>Age: ___ ___ ___
Does the patient comply with inclusion criteria? ☐ Yes ☐ No		

Patient history

Brief description of organic systems:

Patient history

Parameter	Yes	No	Comments
Positive family history	☐	☐	
Diabetes	☐	☐	
Dyslipidemia	☐	☐	
Hypertension	☐	☐	

Smoking	☐	☐	Packs: _______ Years:________
Previous CAD	☐	☐	
Previous CAGB	☐	☐	
Previous PCI	☐	☐	
Heart failure	☐	☐	
History of stroke/TIA	☐	☐	
Erythropoietin therapy	☐	☐	
Atrial fibrillation	☐	☐	
**Duration of dialysis (months)**			
**KT/V**			

Clincal Examination of the patient

Date of examination *(DD/MM/GGGG)*: ___ ___ / ___ ___ / ___ ___ ___ ___		
Hight: __ __ __ . __ __ ☐ cm	Weight: __ __ __ . __ __ ☐ kg	Comments

ECOG Performance Status: ☐ 0 ☐ 1 ☐ 2 ☐ 3 ☐ 4

ECOG PERFORMANCE STATUS*	
**Grade**	**ECOG**
0	Fully active, able to carry on all pre-disease performance without restriction
1	Restricted in physically strenuous activity but ambulatory and able to carry out work of a light or sedentary nature, e.g., light house work, office work
2	Ambulatory and capable of all selfcare but unable to carry out any work activities. Up and about more than 50% of waking hours
3	Capable of only limited selfcare, confined to bed or chair more than 50% of waking hours
4	Completely disabled. Cannot carry on any selfcare. Totally confined to bed or chair
5	Dead

<u>Patients drugs:</u>
